# Metabolism of Phenolic Compounds and Antioxidant Activity in Different Tissue Parts of Post-Harvest Chive (*Allium schoenoprasum* L.)

**DOI:** 10.3390/antiox13030279

**Published:** 2024-02-25

**Authors:** Xiaomei Dai, Chonglei Jia, Jiaqi Lu, Zhifang Yu

**Affiliations:** 1 College of Food Science and Technology, Nanjing Agricultural University, Nanjing 210095, China; 2019208001@njau.edu.cn (X.D.); 2022808133@stu.njau.edu.cn (C.J.); 2021108057@stu.njau.edu.cn (J.L.); 2 Department of Food Science and Technology, Jiangsu Food & Pharmaceutical Science College, Huaian 223003, China

**Keywords:** *Allium* plants, antioxidant, chive, metabolism, phenolic compounds, post-harvest

## Abstract

Chive (*Allium schoenoprasum* L.) has a strong antioxidant property as it contains abundant phenolic compounds and ascorbic acid. In the present study, we investigated the metabolism of phenolic compounds and the change in antioxidant activity in different tissue parts of post-harvest chives. The results showed that compared with the bottom white part (BW), the round green part (RG) exhibited significantly higher contents of phenolic compounds, increased enzyme activities and enhanced antioxidant activities, indicating that phenolic compounds were mainly synthesised in RG. The expression levels of genes such as phenylalanine ammonia–lyase, cinnamate 4-hydroxylase and 4-coumaroyl-CoA ligase and their corresponding enzyme activities rapidly decreased in RG, whereas they were maintained in BW, suggesting that senescence occurred more rapidly in RG than in BW. Our study provides a theoretical basis for further research into and development of different parts of *Allium* plants and offers a basis for consumers’ nutritional considerations.

## 1. Introduction

The genus *Allium*, comprising several hundred species, is an important food plant that is widely utilised as a drug in folk and modern medicine for its anti-microbial, lipid-lowering, cardiovascular, hypocholesterolaemic, anti-thrombotic, hypoglycaemic and anti-tumorigenic activities [1]. The development of associated diseases is believed to be related to oxidative stress. Globally, chive (*Allium schoenoprasum* L.) is cultivated as a vegetable or seasoning herb. Chive is rich in organosulfur compounds, including the flavour precursor S-alk(en)ylcysteine sulfoxides (with isoalliin being the major compound in chive) and their degradative compounds (e.g., thiosulfinates and polysulfides) [2]. However, our previous study on chive [2], using different measurement methods, showed that these compounds exhibited weak antioxidant activities. In contrast, phenolic compounds and ascorbic acid (AsA) were found to significantly contribute to the antioxidant activities in chive.

Phenolic compounds with one or more hydroxyl groups exhibit antioxidant activity, which is closely related to the scavenging of reactive oxygen species and stress response [3]. Studies show that total phenolic content is significantly and positively correlated with antioxidant activity in *A. cepa* and *A. semenovii* [4], *A. scorodoprasum* [5], leek (*A. porrum* L.) [6] and garlic (*A. sativum* L.) [7].

Phenolic compounds were reported to be abundant in chive, and *p*-coumaric acid and ferulic acid were the major phenolics [8,9]. According to the literature [10], chives exhibit the highest antioxidant activity compared with other *Allium* plants (e.g., onion, garlic, shallot, leek and bunching onions). Apart from phenolic compounds, chive also contains an ample amount of AsA, which is highly associated with antioxidant activity [2].

*Allium* species have different tissue parts. For instance, chive, leek, Chinese chives (*Allium tuberosum*) and Welsh onion (*Allium fistulosum* L.) have a round green part (RG) and a bottom white part (BW), whereas garlic and onion have green leaves and cloves or bulbs. Thus, understanding the phytochemical and physiological properties of different tissues of *Allium* plants is crucial for developing and utilising these important medicinal plants. Different tissue parts were proven to possess different concentrations of phenolic compounds in *A. scorodoprasum* [5], *A. galanthum* [11] and *A. ursinum* [12]. The dynamics of phenolic compounds and antioxidant activities were also studied in post-harvest onion [13], garlic [7] and leek [14]. However, information regarding these dynamics in different parts of post-harvest *Allium* plants is scarce. Our previous study showed that a higher content of organosulfur compounds was mainly present in BW in chive, and these compounds were continuously translocated from the predominantly biosynthesised tissue (RG) of chive to BW during post-harvest storage [15,16]. However, the mechanism of the biosynthesis of phenolic compounds that occurs in different parts of *Allium* plants during post-harvest storage is unclear.

This study aimed to accomplish the following: (i) characterise the dynamics of phenolic compounds and antioxidant activities in different parts of post-harvest chive and (ii) elucidate the mechanism underlying the biosynthesis of phenolic compounds in different parts of *Allium* plants during post-harvest storage. Our study will provide new insights into phenolic compound biosynthesis in different tissue parts of post-harvest chive and offer a theoretical basis for further research and development of *Allium* plants, considering different tissues containing various bioactive compounds.

## 2. Materials and Methods

### 2.1. Materials and Treatments

The experimental material chive (*A. schoenoprasum* L. cv. Xinghua), which was purchased from a farmer, was grown in Xinghua County, Jiangsu Province, China. Chives after harvest were transported to the laboratory within 3 h at 20 °C. The soil on the underground part of the chives was washed away. Senescent leaves were removed, and those with uniform sizes were selected and dried to remove the water on the surface of chives using a fan. Every 2 kg of chives were packed in a polyethylene plastic bag (thickness, 0.08 mm) and then stored in the dark at room temperature (RT, 20 ± 0.5 °C) for 5 days or low temperature (LT, 4 ± 0.5 °C) for 19 days with 80–90% relative humidity.

At each time point, 3 kg of chives were sampled in each group and separated into two parts, RG and BW. The RG and BW of the chive were cut into pieces of 0.5–1 cm separately and immediately frozen in liquid nitrogen and stored at −80 °C for further analysis.

The 21 phenolic compound standards (≥98%, HPLC) used for liquid chromatography, including phenylalanine, trans-cinnamic acid, *p*-coumaric acid, caffeic acid, ferulic acid, sinapic acid, *p*-hydroxybenzoic acid, vanillic acid, gallic acid, quercetin, isorhamnetin, dihydrokaempferol, dihydroquercetin, hesperitin, neochlorogenic acid, chlorogenic acid, rutin, apigenin, luteolin, naringenin chalcone and hyperoside, were purchased from Shanghai Yuanye Bio-Technology Co., Ltd. (Shanghai, China). Acetonitrile and acetic acid (≥99%, HPLC) were purchased from Sigma-Aldrich (Shanghai, China). Chemical reagents, including Folin–Ciocalteu reagent, 1,1-diphenyl-1-picrylhydrazyl (DPPH), 6-hydroxy-2,5,7,8-tetramethylchroman-2-carboxylic acid (Trolox), 2,4,6-tri(2-pyridyl)-s-triazine (TPTZ), AsA, FeCl_3_·6H_2_O, FeSO_4_·7H_2_O, salicylic acid, pyrogallol, ethanol, sodium hydroxide and hydrochloric acid were of analytical grade and purchased from Sigma-Aldrich (Shanghai, China).

### 2.2. Preparation of Extracts

The samples were frozen with liquid nitrogen and ground into a powder. A 1 g sample was extracted with 5 mL of ethanol/water mixture (70/30, *v*/*v*) for 1 h at 35 °C in a water bath, with continuous shaking at 120 rpm. The mixture was centrifuged at 12,000× *g* for 10 min at 4 °C, and the supernatant was collected. The residue was extracted with another 3 mL of ethanol/water mixture (70/30, *v*/*v*) under the same conditions. Subsequently, the supernatants were combined. These extracts were prepared for Folin–Ciocalteu assay and antioxidant activity assessment.

The extracts used to detect the phenolics (including free and bound phenolics) content were prepared using a method described previously [17], with slight modifications. The sampled RG and BW, which were stored at −80 °C previously, were ground into powder in liquid nitrogen separately. Then, 5 g of RG or BW sample powder was weighed into a 250 mL flask, and 25 mL of 2 M hydrochloric acid was added. The flask was covered with a plastic sealing film tightly and placed in a water bath pot and incubated for 1.5 h at 80 °C under dark conditions, with the speed of the oscillator maintained at 100 rpm. After cooling, the sample extract was adjusted to pH 2 with 6 N NaOH. Phenolic compounds were extracted thrice with 20 mL of ethyl acetate; each extracted sample was placed in a shaker at 250 rpm and 25 °C for 20 min under dark conditions. The combined extracts were evaporated to obtain 3–5 mL of sample, which was transferred to a 10 mL tube and completely dried with nitrogen. Subsequently, 1 mL of methanol was added to the tube to dissolve the phenolic compounds. The extract was filtered and subjected to ultrahigh-performance liquid chromatography coupled with tandem mass spectrometry (UHPLC-MS/MS) to test the phenolic compounds.

### 2.3. The Folin–Ciocalteu Assay and Detection of AsA

Total phenolic content was measured using the Folin–Ciocalteu method (TPC-FC) described by Dai et al. [2]. Absorption was measured at 760 nm against a blank, and the amount of TPC-FC was expressed as gallic acid equivalents (mg gallic acid/g sample) through the calibration curve of gallic acid.

To determine AsA content, 1 g of chive powder ground with liquid nitrogen was weighed into a tube, after which 10 mL of 5% (*w*/*v*) trichloroacetic acid was added and mixed. Then, the mixture was centrifuged at 12,000× *g* for 15 min at 4 °C. The supernatant was collected to determine the AsA content, which was measured following a method described previously [18].

### 2.4. UHPLC-MS/MS Method

Phenolic compounds were measured using the UHPLC-MS/MS method reported by Dai et al. [2]. These compounds were quantified using a model Exion LC system (AB SCIEX, Framingham, MA, USA), which was interfaced with a Triple Quad 5500 mass spectrometer (AB SCIEX, Framingham, MA, USA) equipped with a Turbo electrospray ion source. Instrument control and data acquisition were conducted using Analyst LC/MS Data Acquisition software version 1.7 (AB SCIEX, Framingham, MA, USA). Data evaluation was performed using SCIEX OS Qualitative and Quantitative Analysis software version 3.0 (AB SCIEX, Framingham, MA, USA). The content of each phenolic compound was expressed as mg kg−1 based on fresh weight, according to their calibration curves (Table S3).

A Kinetex F5 LC column (2.1 × 100 mm, 2.6 μm, 100 Å) (Phenomenex, Torrance, CA, USA) was used to separate the phenolic compounds. Further, 0.2% acetic acid in distilled water (solvent A) and acetonitrile containing 0.2% acetic acid (solvent B) were used as the mobile phase, with a flow rate of 0.15 mL min^−1^. The gradient profile was as follows: 2–70% B (0–12 min), 70–95% B (12–12.5 min), 95–95% B (12.5–13.5 min), 95–2% B (13.5–14 min), 2–2% B (14–17 min). The injection volume was 1 μL, and the column temperature was maintained at 40 °C. The LC effluent was connected to the interface through a divert valve to prevent clogging of the mass spectrometer cone.

Mass spectrometry employed electrospray ionisation in the negative ion mode. Multiple reaction monitoring modes were used to monitor precursor–product ion transitions and conduct quantification, with corresponding parameters detailed in Table S1. The general settings employed included collision gas pressure of 8 psi, curtain gas pressure of 35 psi, ion source gas (1) pressure of 55 psi, ion source gas (2) pressure of 55 psi, ion spray voltage of 5500 V and temperature of 550 °C.

### 2.5. Determination of Enzyme Activities Involved in Phenolic Compounds Biosynthesis and Degradation

Frozen powder (1 g) was homogenised using 5 mL of 100 mM phosphate buffer (pH 6.0) and 2% polyvinylpolypyrrolidone, then centrifuged at 10,000× *g* for 30 min at 4 °C. The activities of phenylalanine ammonia–lyase (PAL), cinnamate 4-hydroxylase (C4H) and 4-coumaroyl-CoA ligase (4CL) were determined using ELISA kits (Mallbio, Nanjing Mallbio Biological Technology Co., Ltd., Nanjing, China), following the methods described by Olivares et al. [19]. The absorbance readings of the reaction mixtures were taken at 290 nm (PAL), 333 nm (4CL) and 340 nm (C4H), with one unit (U) of enzyme activity defined as a 0.01 increase in absorbance per hour per gram.

Peroxidase (POD) and polyphenol oxidase (PPO) activities were determined following the method proposed by Zhang et al. [3]. One unit of POD and PPO activity was defined as an increase of 1 in absorbance per minute per gram at 460 nm and 420 nm, respectively. Units for all enzymes were expressed as U g^−l^ based on fresh weight.

### 2.6. Determination of Relative Gene Expression

Real-time quantitative PCR (qPCR) was used to test the relative expression levels of the targeted genes in the phenylpropanoid metabolism pathway in RG and BW. Twenty-three unigenes associated with the phenylpropanoid metabolism pathway were analysed using an SYBR Premix Ex Taq II (TaKaRa, Dalian, China) kit, and the reaction was conducted using the StepOne Real-Time PCR System (Applied Biosystems, Foster City, CA, USA). Three replicates were conducted, and the relative expression levels of the selected unigenes were normalised (with the *ACTIN* gene as an internal control) and determined using the 2^−ΔΔCt^ method. The primers used for qPCR analysis are presented in Supplementary Table S2.

### 2.7. Determination of Antioxidant Activities

We followed the procedures described by Dai et al. [2] to determine the ferric-reducing antioxidant power (FRAP), scavenging activity towards 1,1-diphenyl-1-picrylhydrazyl (DPPH), superoxide anion (O_2_^•−^) and hydroxyl radical (^•^OH). Absorbance was measured at 593 nm (FRAP), 517 nm (DPPH), 510 nm (^•^OH) and 320 nm (O_2_^•−^). The relative ^•^OH and O_2_^•−^ scavenging capacity of the chive extracts were expressed in milligrams of AsA equivalents per gram of fresh weight (mg VE g^−1^). The relative FRAP capacity and DPPH scavenging capacity of the chive extracts were expressed in micromoles of Trolox equivalents per gram of fresh weight (µmol TE g^−1^).

### 2.8. Statistical Analysis

All values are reported as the mean ± standard errors of three biological replicates. Statistical analysis was performed with SPSS Statistics 27 software (SPSS Inc, Chicago, IL, USA). The significant differences between mean values were analyzed using the Duncan’s multiple range test (*p* < 0.05). Origin 2021 (Microcal Software, Northampton, MA, USA) and Adobe Illustrator 2020 software (Adobe Inc., San Jose, CA, USA) was employed to create figures.

## 3. Results and Discussion

### 3.1. Principal Component Analysis

**Figure 1:**
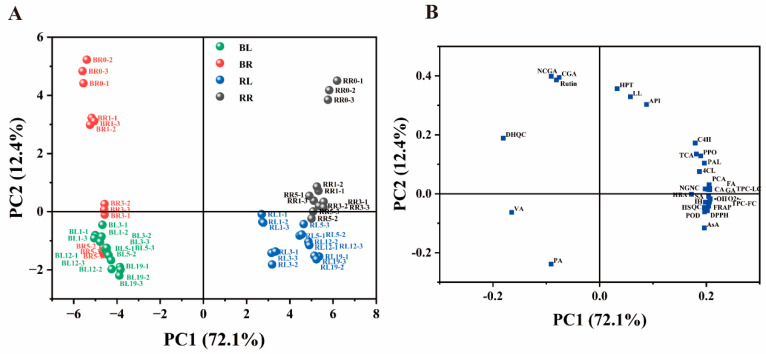
(**A**) Scatter plot of chive samples and (**B**) component loading plot of 32 parameters (including phenolic compounds, ascorbic acid, related enzymes and antioxidant parameters) obtained through principal component analysis. RL, round green part (RG) stored at 4 °C. BL, bottom white part (BW) stored at 4 °C; RR, RG stored at 20 °C. BR, BW stored at 20 °C. PA, phenylalanine; TCA, trans-cinnamic acid; PCA, *p*-coumaric acid; CA, caffeic acid; FA, ferulic acid; SA, sinapic acid; HBA, p-hydroxybenzoic acid; VA, vanillic acid; GA, gallic acid; QC, quercetin; IH, isorhamnetin; DHQC, dihydroquercetin; HPT, hesperitin; NCGA, neochlorogenic acid; CGA, chlorogenic acid; RUT, rutin; API, apigenin; LL, luteolin; NGNC, naringenin chalcone; TPC-FC, total phenolic content measured using the Folin–Ciocalteu method; TPC-LC, sum of phenolic compounds measured via LC–MS; AsA, ascorbic acid.

In this study, principal component analysis was performed to visualise the variation and correlation among 20 individual phenolic compounds, AsA content, antioxidant activities and the activities of the enzymes involved in the biosynthesis and degradation of phenolic compounds in different parts of chive during storage (Figure 1). The loading plot (Figure 1B) shows that AsA, antioxidant indexes, enzyme indexes and most of the phenolic compounds (trans-cinnamic acid (TCA), *p*-coumaric acid (PCA), ferulic acid (FA), caffeic acid (CA), gallic acid (GA), *p*-hydroxybenzoic acid (HBA), sinapic acid (SA), isorhamnetin (IH), quercetin (QC), vanillic acid (VA), dihydroquercetin (DHQC), naringenin chalcone (NGNC) and the sum of total phenolic compounds (TPC-LC)) significantly contributed to PC1. In contrast, neochlorogenic acid (NCGA), chlorogenic acid (CGA), rutin, hesperetin (HPT), luteolin (LL) and apigenin (API) significantly contributed to PC2. PC1 mainly differentiated between the RG and BW groups, indicating that tissue specificity was the major contributor to clustering. PC2 primarily differentiated between the RT and LT groups. The RG group exhibited a higher content of most phenolic compounds, AsA, antioxidant activities and enzyme activities compared with those in the BW, whereas the BW group exhibited a higher content of DHQC, VA and phenylalanine (PA).

### 3.2. Dynamics of Antioxidant Compounds

#### 3.2.1. Dynamics of TPC-FC and AsA

**Figure 2:**
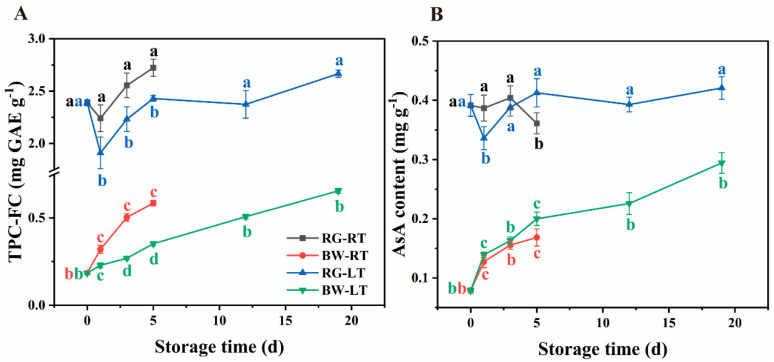
Contents of (**A**) TPC-FC and (**B**) ascorbic acid in different parts of chives during storage at 20 °C (RT) and 4 °C (LT). RG, round green part. BW, bottom white part. Data are presented as the means of three independent measurements. The letters represent differences among different tissues under RT and LT at each time point.

In post-harvest chive, the TPC-FC content in RG and BW was 1.91–2.72 and 0.19–0.66 mg GAE g^−1^, respectively. The TPC-FC content was 3- to 12-fold higher in RG than in BW during storage (Figure 2A, Table S4). In RG, TPC-FC declined on day 1 and gradually increased thereafter under room and low temperatures, with 10.77–14.75% higher content observed in chives under RT than under LT. In BW, the TPC-FC content continuously increased during storage, with 28.59–46.45% higher content observed in chives under RT than under LT.

The AsA content in RG and BW was 0.34–0.42 and 0.08–0.29 mg g^−1^, respectively. The AsA content was 1.1- to 4.0-fold higher in RG than in BW (Figure 2B, Table S5). In RG, the AsA content decreased on day 5 under RT, whereas it decreased on day 1 under LT, followed by an increase. Furthermore, in RG, the AsA content was 15.13% higher on day 1 but 12.51% lower on day 5 than those under LT. Conversely, in BW, the AsA content tended to rise gradually under both temperatures, with no significant difference between the content under RT and LT.

#### 3.2.2. Dynamics of Phenolic Compounds Determined Using LC–MS

**Figure 3:**
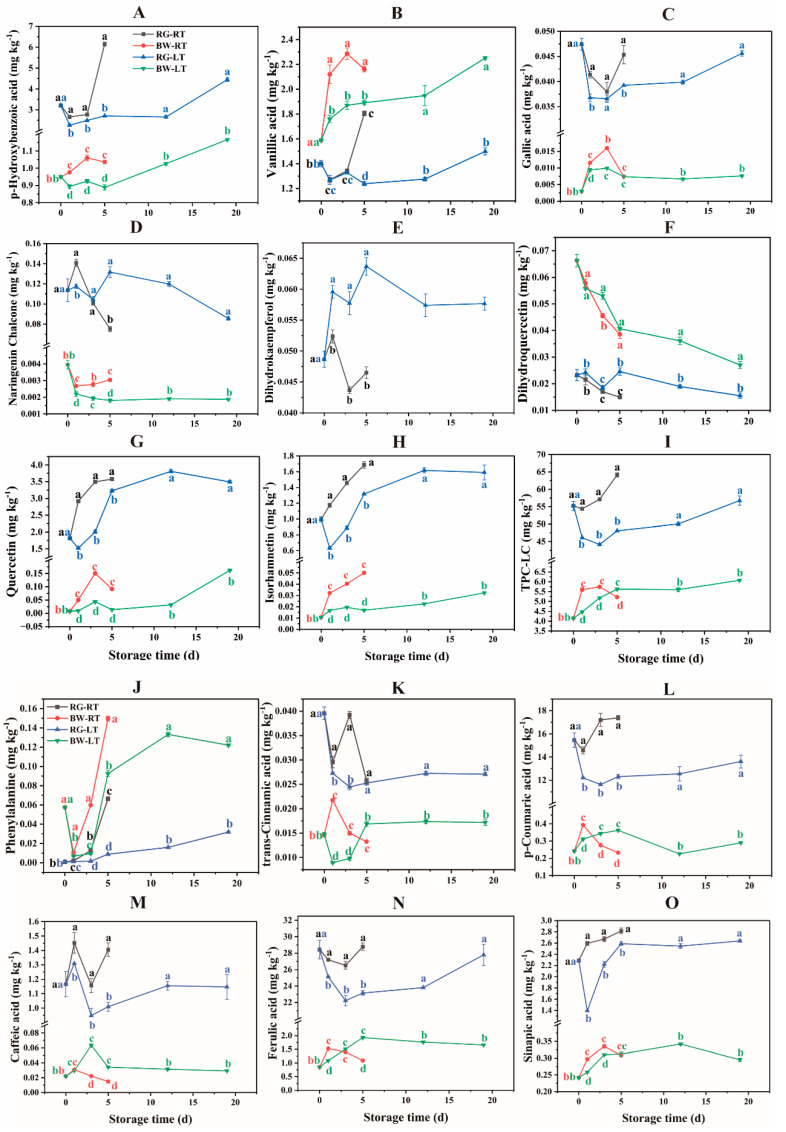
Phenolic compound profiles in different parts of chive stored at 20 °C (RT) and 4 °C (LT). RG, round green part. (**A**) p-hydroxybenzoic acid, (**B**) vanillic acid, (**C**) gallic acid, (**D**) naringenin chalcone, (**E**) dihydrokaempferol, (**F**) dihydroquercetin, (**G**) quercetin, (**H**) isorhamnetin, (**I**) TPC-LC, (**J**) phenylalanine, (**K**) trans-cinnamic acid, (**L**) *p*-coumaric acid, (**M**) caffeic acid, (**N**) ferulic acid, (**O**) sinapic acid. BW, bottom white part. Data are presented as the means of three independent measurements. The letters represent differences among different tissues under RT and LT at each time point.

Twenty-one phenolic compounds were detected in the chives (Figure 3 and Table S6), and the LC–MS chromatograph is shown in Figure S1. Regarding RG, FA (22.22–28.79 mg kg^−1^) and PCA (11.63–17.40 mg kg^−1^) were the most abundant, followed by HBA (2.26–6.15 mg kg^−1^), QC (1.52–3.81 mg kg^−1^), SA (1.39–2.81 mg kg^−1^), VA (1.24–1.80 mg kg^−1^), IH (0.63–1.69 mg kg^−1^) and CA (0.95–1.45 mg kg^−1^). For BW, VA (1.60–2.30 mg kg^−1^), FA (0.85–1.93 mg kg^−1^) and HBA (0.90–1.16 mg kg^−1^) were the major phenolic compounds. Rutin, HPT, API and LL were detected in trace amounts in chives. The TPC-LC content in RG and BW was 44.16–64.14 mg kg^−1^ and 4.15–6.07 mg kg^−1^, respectively, indicating that the content of phenolic compounds was much higher in RG than in BW.

Currently, FA is utilised as an additive antioxidant with pharmacological applications for human health [6]. In this study, FA and PCA were predominant in chives, consistent with a previous study [8]. It appeared that different phenolic compounds dominated in different *Allium* species, for example, GA in *A. stylosum* [20], PCA in *A. subhirsutum* [21], protocatechuic acid and VA in *A. semenovii* [4], FA in leek [6] and QC in onion [22], were reported as the most dominant phenolic compounds in these species.

HBA, VA and GA, which are hydroxybenzoic acids, were detected in chives. HBA content in RG was 1.62- to 4.94-fold and 1.52- to 2.81-fold higher than those in BW under RT and LT, respectively. HBA content increased in chives under both temperatures. The HBA content in RG was higher under RT than under LT, especially on day 5, with a 127.15% increase in content. The HBA content in BW was 9.19–16.64% higher under RT than under LT. VA is a natural antioxidant present in fruits and vegetables [23]. Unlike other phenolic compounds, VA content was observed to be 13.35–70.23% and 13.35–52.75% higher in BW than in RG under RT and LT, respectively. VA content increased in BW during storage, with a 14.34–22.27% higher content under RT compared with that at LT. In RG, VA content increased during the late storage stage, with a 45.66% higher content under RT than that under LT on day 5. GA was 1.37- to 15.07-fold and 2.68- to 15.07-fold higher in RG than in BW under RT and LT, respectively. HBA, VA and GA content were lower in chives under LT than under RT, suggesting that LT suppressed hydroxybenzoic acid biosynthesis in RG and BW.

PA is the initial substance in the phenylpropanoid pathway and can be catalysed into phenolic acids [24]. The concentration of PA in RG and BW increased continuously during storage, with a more pronounced increase in BW than in RG under both temperatures. The PA content was higher in both RG and BW under RT than under LT. The TCA content in RG was 35.78–168.96% and 49.72–205.00% higher than in BW under RT and LT, respectively. The TCA content in RG was 8.36% and 60.35% higher under RT than under LT on days 1 and 3, respectively. The TCA content in BW was 143.40% and 52.79% higher under RT than under LT on days 1 and 3, respectively. The PCA content was 36.09- to 73.70-fold and 32.83- to 62.86-fold higher in RG than in BW under RT and LT, respectively. Furthermore, in RG, the PCA content increased under RT but declined under LT. The PCA content in RG was 19.46–47.88% higher under RT than under LT. Conversely, in BW, PCA content initially increased and then decreased under both temperatures. Additionally, PCA content in BW was higher on day 1 but lower on day 3 and 5 under RT compared with LT.

NGNC is a key compound for the biosynthesis of downstream flavonoids. NGNC content was 23.68- to 51.69-fold and 27.85- to 71.75-fold higher in RG than in BW under RT and LT, respectively. NGNC content in RG was higher on day 1 but lower on day 5 under RT compared with LT. NGNC content in BW was higher under RT than under LT. Moreover, dihydrokaempferol (DHKF) content in RG was 13.74–36.99% higher under LT than under RT. However, DHKF was not detected in BW. DHQC content was 158.24–184.81% and 65.38–186.35% higher in BW than in RG under RT and LT, respectively. DHQC content in RG and BW showed a gradually decreasing trend under both temperatures, with a more rapid decline under RT than under LT. NGNC is a precursor of DHKF, which is further converted to DHQC in the phenylpropanoid pathway [25]. NGNC content was significantly lower in BW than in RG, whereas DHQC content was higher in BW than in RG. These results suggest that flavonoids are rapidly biosynthesised in BW during storage.

QC, the main flavonoid detected, was 22.22- to 222.81-fold and 20.63- to 235.98-fold higher in RG than in BW under RT and LT, respectively. In both RG and BW, QC increased under both temperatures. The QC content in RG was 10.93–92.06% higher under RT than under LT. The QC content in BW was 244.53–572.83% higher under RT than under LT. IH content was 32.69- to 91.85-fold and 36.90- to 91.85-fold higher in RG than in BW under RT and LT, respectively. IH showed an increasing trend in RG and BW, except for a decline observed on day 1 in RG under LT. The IH content in RG was 28.02–85.47% higher under RT than under LT. The QC content in BW was 92.18–195.51% higher under RT than under LT.

Flavonoids are important antioxidant compounds that protect fruits and vegetables against oxidative damage throughout the post-harvest period [23]. The observed higher content of QC and IH in RG than in BW was in agreement with a previous study reporting the green shaft of leek contained a higher flavonoid content than the white shaft [6]. Moreover, the increased content (the difference in the values obtained at the end of storage and day 0) of QC and IH was much higher in RG than that in BW under both temperatures, suggesting that flavonoids were mainly biosynthesised in RG during storage. However, notably, the increased percentage ((value obtained at the end of storage − value obtained on day 0)/value obtained on day 0) of flavonoids was significantly higher in BW than in RG. The increased percentage of QC in RG was 97.01% and 92.33% under RT and LT, respectively, whereas in BW, it was 1028.57% and 1890.39% under RT and LT, respectively. Similarly, the percentage increase of IH in RG was 69.28% and 59.74% under RT and LT, respectively, whereas in BW, it was 366.50% and 201.37% under RT and LT, respectively. Furthermore, the flavonoid content in chives was higher under RT than under LT, indicating that LT suppressed their biosynthesis.

CA content was 46.06- to 93.38-fold and 13.95- to 51.63-fold higher in RG than in BW under RT and LT, respectively. However, CA content in RG was observed to fluctuate during storage, with 10.84–39.11% higher content under RT than under LT. In contrast, CA content in BW initially increased and then declined under both temperatures, with 186.36% and 126.67% higher content under LT than under RT on days 3 and 5, respectively. This result was contrary to that observed in RG. FA was 16.80- to 32.42-fold and 11.01- to 32.42-fold higher in RG than in BW under RT and LT, respectively. In RG, FA decreased initially and then increased under both temperatures, with 8.28–24.35% higher content observed under RT than under LT. Conversely, FA in BW initially increased and then declined. FA in BW under RT was higher on day 1 but was lower on day 5 as compared with that under LT. SA in RG was 6.98- to 8.46-fold and 4.39- to 8.46-fold higher than in BW under RT and LT, respectively. In RG, SA showed a gradual increase under RT, whereas it sharply dropped on day 1 under LT and then increased to the initial value on day 5. SA in RG was higher under RT than under LT, especially on day 1, with an 86.26% higher content. In BW, SA initially increased and then decreased during the late storage stage, with a higher content under RT than under LT.

TPC-LC in RG was 8.71- to 12.32-fold and 7.52- to 12.32-fold higher than in BW under RT and LT, respectively. In RG, TPC-LC increased under RT, whereas it initially decreased and then gradually increased from day 3 till the end of storage under LT. TPC-LC in RG was 17.92–33.37% higher under RT than under LT. In BW, TPC-LC increased under both temperatures, with a 10.85–25.36% higher content under RT on days 1 and 3 but a lower content on day 5 compared with that under LT.

Hydroxycinnamic acids, such as PCA, CA, FA and SA, are important antioxidants and precursors of monolignols, directly linked to lignin biosynthesis [3,26]. As TCA, PCA, CA and FA decreased on day 1 and/or 3 in RG, a corresponding increase occurred in BW. These phenolic acids, along with SA and TPC-LC in RG, exhibited lower content under LT than under RT, suggesting that LT inhibited their biosynthesis. However, in BW, some of these compounds (e.g., PCA and CA on days 3 and 5 and FA on day 5) were higher under LT than under RT. Additionally, the increased content of these compounds in RG was significantly higher under RT than under LT, including FA (0.36, −0.63 mg kg^−1^), PCA (1.93, −1.85 mg kg^−1^), HBA (2.94, 1.24 mg kg^−1^), VA (0.40, 0.10 mg kg^−1^), total phenolic acids (6.46, −0.81 mg kg^−1^) and TPC-LC (8.90, 1.48 mg kg^−1^). However, the increased levels of these compounds in BW were much less under RT than under LT for FA (0.24, 0.81 mg kg^−1^), PCA (−0.01, 0.05 mg kg^−1^), HBA (0.09, 0.22 mg kg^−1^), VA (0.57, 0.66 mg kg^−1^), total phenolic acids (1.04, 1.85 mg kg^−1^) and TPC-LC (1.07, 1.93 mg kg^−1^). The biosynthesis of phenolic compounds in RG was inhibited under LT. Conversely, BW showed a considerably increased content of phenolic acids under LT than under RT. A possible explanation is that LT maintained the quality of chive and promoted the transfer of phenolic compounds from RG to BW.

The inhibitory effect of LT on phenolic compounds in post-harvest chives aligns with previous studies, which reported reduced anthocyanin content in blood oranges stored at 4 °C compared with 9 °C [27]. This alignment is further supported by the continuous accumulation of anthocyanin and phenolic compounds in Purple-Pericarp Supersweet Sweetcorn during storage at 23 °C while remaining unchanged at 4 °C [28] and the lower content of phenolic compounds in mango fruits stored at 5 °C compared with those stored at 13 °C [29].

Overall, the content of most phenolic compounds in RG was significantly higher than that in BW, except for VA, PA, DHQC, CGA and NCGA. The difference in the composition and concentration of phenolic compounds between RG and BW aligns with several previous studies reporting (1) the bulb extract of *A. scorodoprasum* as inferior in phenolic compounds compared with the flower and stem [5], (2) a much higher amount of the major phenolic kaempferol in leaves than in the bulb of *Allium galanthum* [11], (3) higher phenolic content in leaves than in bulb extracts of *A. ursinum* [12] and (4) the green shaft of leek containing a higher content of phenolic acids compared with that in the white shaft [6].

### 3.3. Dynamics of Enzyme Activities Involved in the Metabolism of Phenolic Compounds in Post-Harvest Chive

**Figure 4:**
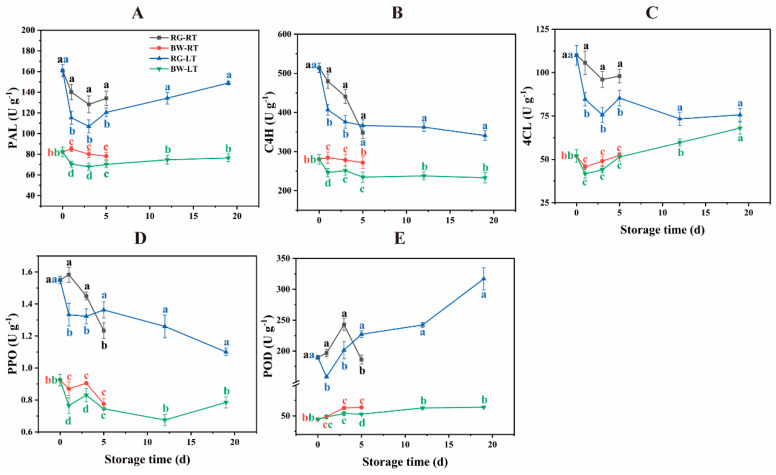
Activities of the enzymes involved in the biosynthesis and degradation of phenolic compounds in different parts of chive stored at 20 °C (RT) and 4 °C (LT). (**A**) PAL, (**B**) C4H, (**C**) 4CL, (**D**) PPO, (**E**) POD. PAL, phenylalanine ammonia–lyase; C4H, cinnamate 4-hydroxylase; 4CL, 4-coumaroyl-CoA ligase; POD, peroxidase; PPO, polyphenol oxidase. RG, the round green part. BW, the bottom white part. Data are the means of the three independent measurements. Letters represent differences among different tissues under RT and LT at each time point.

Generally, phenylpropanoid biosynthesis initiates with the deamination of phenylalanine by PAL, yielding cinnamic acid and downstream phenylpropanoids [23]. C4H catalyses the conversion of TCA to PCA [30]. Furthermore, 4CL catalyses the conversion of PCA to *p*-coumaroyl CoA, a central intermediate for various phenylpropanoid metabolites [30]. PAL activity in RG was 57.39–95.30% higher than in BW (Figure 4A). The PAL activity was 11.34–21.62% higher in RG under RT than under LT. The PAL activity was 11.40–20.70% higher in BW under RT than under LT on days 1 and 3. C4H activity in RG was 28.21–83.42% higher than that in BW (Figure 4B). On days 1 and 5, C4H activity was 18.03% and 17.30% higher in RG under RT than under LT and 15.43% and 15.77% higher in BW under RT than under LT, respectively. 4CL activity in RG was 11.28–131.38% higher than in BW (Figure 4C). In RG, 4CL activity was 14.85–24.80% higher under RT than under LT, with no significant difference observed between both temperatures in BW. The activities of these enzymes in RG significantly declined in the first three days, exhibiting slight changes in BW during the same period. These findings suggest that physiological changes occurred more rapidly in RG compared with BW, indicating faster senescence in RG.

The main enzymes involved in the catabolism of phenolic compounds in fruits and vegetables are PPO and POD [23]. POD is involved in the final step of monolignol polymerisation, forming lignin and eliminating H_2_O_2_ [3]. The PPO activity was 40.13–86.67% higher in RG than in BW (Figure 4D). This activity decreased in chives under both temperatures, with the activity being 18.75% and 9.60% higher on days 1 and 3 but 9.54% lower on day 5 in RG under RT compared with that under LT. The PPO activity in BW was 13.73% and 9.04% higher under RT than under LT on days 1 and 3, respectively. The POD activity was 1.9- to 3.9-fold higher in RG than in BW (Figure 4E). Moreover, it increased in chives under both temperatures, with the exception of the decrease observed in RG on day 5 under RT. On days 1 and 3, the activity was 24.33% and 20.56% higher in RG under RT but 18.12% lower on day 5 compared with that observed under LT. Conversely, it was 20.92% higher on day 5 in BW under RT than under LT.

The enzymes associated with the biosynthesis and degradation of phenolic compounds showed higher activities in RG compared with BW. LT inhibited both the biosynthesis and degradation of phenolic compounds in post-harvest chives. This finding is consistent with reduced PAL activity in sweet cherry stored at LT (1 °C) compared with RT (20 °C) [31] and lower POD activity in chives stored at 3 °C compared with 20 °C [32].

### 3.4. Dynamics of Gene Expression

**Figure 5:**
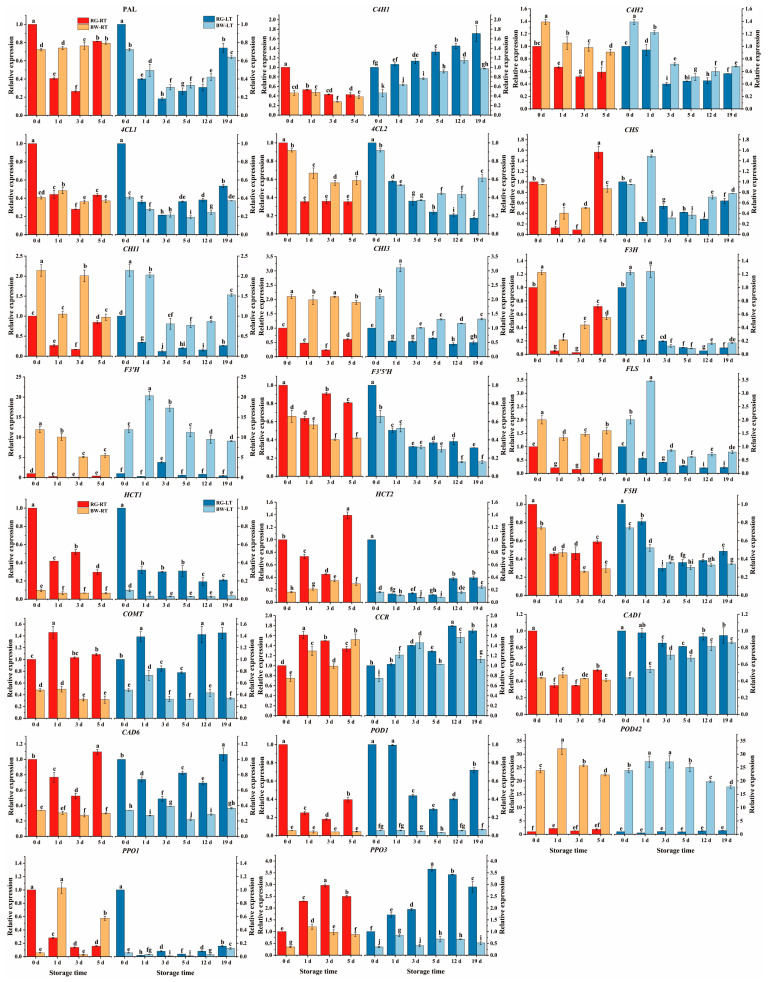
Expression pattern of genes (qPCR) involved in the biosynthesis and degradation of the phenolic compounds present in different parts of chive stored at 20 °C (RT) and 4 °C (LT). RG, round green part. BW, bottom white part. Data are presented as the means of three independent measurements. The letters represent statistical differences among different time points and tissues under RT or LT.

As shown in Figure 5, the relative expression of *PAL* in RG under both RT and LT initially declined and then increased during the late storage stage. Conversely, *PAL* in BW remained constant under RT but declined initially and then increased under LT. *PAL* generally maintained a higher level of expression in BW than in RG during storage, with a 0.82- and 1.89-fold increase in expression levels on days 1 and 3 under RT and 0.23- to 0.70-fold higher expression from days 1–12 under LT. LT inhibited *PAL* expression during storage. *C4H1* in chives showed different trends under RT and LT, decreasing under RT but increasing under LT. Its expression was higher in RG compared with BW under LT, showing a 0.26- to 0.76-fold increase. Conversely, *C4H2* showed a declining trend, with elevated expression in BW than in RG, with a 0.54- to 0.90-fold increase under RT and a 0.15- to 0.80-fold increase under LT. LT inhibited *C4H2* expression on days 3 and 5 compared with RT. Both *4CL1* and *4CL2* showed decreasing trends during storage. BW showed a higher expression level in *4CL2* than RG, with a 0.56- to 0.88-fold increase under RT and a 1.85- to 3.57-fold increase from days 5 to 19 under LT. Notably, LT inhibited the expression of *4CL1* and *4CL2*.

*PAL*, *C4H2* and *4CL2* exhibited higher expression levels in BW than in RG post-harvest, indicating that BW maintained gene expression in the phenylpropanoid pathway. LT almost suppressed the expression of *PAL*, *C4H2*, *4CL1* and *4CL2*, indicating that LT inhibited the pathway. These findings align with previous studies reporting that LT suppresses the expression levels of genes related to phenolic compound biosynthesis in sweet cherry [31] and blood orange [27].

*CHS* converts 4-coumaroyl-CoA to naringenin chalcone, which is further converted to naringenin by *CHI* [33]. Naringenin, which contains a flavonoid skeleton, can be modified and transformed into different flavonoids (e.g., DHQC, DHKF, QC and IH) [3] by several enzymes, including flavanone 3-hydroxylase (*F3H*), flavonoid 3′-hydroxylase (*F3*′*H*), flavonoid 3′,5′-hydroxylase (*F3*′5′*H*) and flavonol synthase (*FLS*).

*CHS* in chives initially decreased and then increased at the later stage of storage under both temperatures, with significantly higher expression in BW on days 1 and 3 under RT (2.19-fold and 4.66-fold) and on days 1 and 12 under LT (5.35-fold and 1.45-fold) compared with that in RG. *CHI1* and *CHI3* in chives under both temperatures decreased, except *CHI3* in BW under RT, which remained constant. *CHI1* exhibited significantly higher levels in BW than in RG under both temperatures, with a 2.93- to 10.90-fold increase on days 1 and 3 under RT and a 2.83- to 5.85-fold increase during LT storage. *CHI3* also showed significantly higher levels in BW than in RG under both temperatures, with a 2.1- to 7.9-fold increase under RT and a 0.89- to 4.69-fold increase under LT. LT increased the expression of *CHI1* on day 1 but decreased its expression on days 3 and 5. Additionally, LT increased *CHI3* expression on day 1 in BW but decreased its level on days 3 and 5 in BW.

*F3H* in chives initially declined and then increased under RT, whereas it gradually decreased under LT. BW exhibited a significantly higher expression level of *F3H* on days 1 and 3 under RT (3.26- and 16.43-fold) and on day 1 under LT (4.79-fold) compared with that in RG. LT suppressed its expression in RG and BW on day 1 but enhanced *F3H* expression in BW on day 3 and in both RG and BW on day 5. *F3*′*H*, responsible for DHQC synthesis, exhibited decreased expression levels in chives at both temperatures. *F3*′*H* showed significantly higher expression levels in BW than in RG, with a 13.00- to 45.32-fold increase under RT and a 3.55- to 68.20-fold increase under LT. Moreover, LT promoted *F3*′*H* expression. Generally, the expression levels of *F3*′5′*H* in chives showed a declining trend under both temperatures. RG exhibited a higher expression of *F3*′5′*H* during the late storage stage under RT and LT compared with that in BW. LT inhibited *F3*′5′*H* expression during storage. *FLS* expression showed a declining trend in chives under both temperatures. *FLS* was expressed more in BW than RG, with a 1.91- to 8.86-fold increase under RT and a 1.08- to 5.16-fold increase under LT. The *FLS* expression level was higher on day 1 in both RG and BW under LT than under RT. The *FLS* expression level in BW was higher on days 3 and 5 under RT than under LT.

*CHS*, *CHI1*, *CHI 3*, *F3H*, *F3*′*H* and *FLS* showed higher expression levels in BW than in RG during storage, indicating that the genes in BW related to flavonoid biosynthesis were more active than those in RG. LT generally increased the expression of *CHS*, *CHI1*, *CHI3*, *F3H* and *FLS* in RG and BW on day 1 but suppressed them on days 3 and/or 5. These findings were consistent with previous studies reporting (1) a lower expression level of genes in the flavonoid pathway in blood oranges during cold storage at 4 °C than at 9 °C [27] and (2) the reduced transcription of genes involved in anthocyanin biosynthesis in sweet cherry during cold storage compared with room storage [31].

The genes coding shikimate O-hydroxycinnamoyltransferase (*HCT1* and *HCT2)* in chives decreased during storage at both temperatures, except for the stable expression of *HCT1* in BW under RT and an increase in *HCT2* expression in BW under RT. *HCT1* demonstrated significantly higher expression levels in RG than in BW, with a 3.51- to 6.67-fold increase under RT and a 4.67- to 9.23-fold increase under LT. The expression level of *HCT2* was generally higher in RG than in BW, with a 0.30- to 3.70-fold increase under RT and a 0.18- to 1.34-fold increase under LT. LT inhibited *HCT1* and *HCT2* expression in RG and BW during storage. The gene coding ferulate 5-hydroxylase (*F5H*) in chives declined during RT and LT storage, with a higher expression level in RG than in BW. LT increased *F5H* in RG on day 1 but decreased its expression on days 3 and 5. Under both temperatures, 3-O-methyltransferase (*COMT*) in chives initially increased and then declined, except for a fluctuation in RG under LT. *COMT* expression was higher in RG than in BW, with a 1.95- to 2.42-fold increase under RT and a 0.90- to 3.24-fold increase under LT. Notably, LT increased *COMT* expression in BW on day 1 but suppressed its expression in RG on days 3 and 5.

Cinnamoyl CoA reductase (*CCR*) is the primary enzyme responsible for producing monolignols from phenylpropanoid metabolites in chives. Generally, *CCR* exhibited an increasing trend under RT and LT conditions during storage, with higher expression in RG than in BW, a 0.25- and 0.51-fold increase on days 1 and 3 under RT and a 0.15- to 0.50-fold increase from days 5 to 19 under LT. Cinnamyl alcohol dehydrogenase (*CAD*) catalyses the final step in the monolignol pathway. Overall, RG showed a higher expression level of *CAD1* compared with that in BW, with a 0.15- to 0.81-fold increase from days 1 to 12 under LT. *CAD6* expression was significantly higher in RG than in BW, with a 0.93- to 2.67-fold increase during RT storage and a 0.25- to 2.80-fold increase during LT storage. LT inhibited *CAD6* expression in chives on day 5.

*POD1* expression in RG declined at both temperatures, whereas it remained largely unchanged in BW during storage. A significantly higher expression of *POD1* was observed in RG than in BW, with a 3.43- to 8.10-fold increase under RT and a 6.33- to 16.59-fold increase under LT. LT induced its expression in RG on days 1 and 3. *POD42* expression increased under both temperatures, with a higher expression level in BW than in RG, showing a 10.80- to 19.12-fold increase under RT and an 11.84- to 50.84-fold increase under LT. This result indicates that *POD1* dominated in RG, whereas *POD42* dominated in BW. *PPO1* in RG showed a declining trend under RT and LT during storage, whereas it fluctuated in BW under RT and decreased in BW under LT. The expression of *PPO3* in RG and BW exhibited an increasing trend under RT and LT during storage, with a significantly higher expression in RG than in BW, showing a 0.90- to 2.0-fold increase under RT and a 1.02- to 4.51-fold increase under LT. LT inhibited the expression of *PPO1* and *PPO3* in RG and BW.

RG showed higher expression levels of *HCT1*, *HCT2*, *F5H*, *COMT, CAD1*, *CAD6*, *POD1* and *POD6* than BW, which are related to lignin biosynthesis [34], indicating that the genes related to lignin biosynthesis were more active in RG than in BW. Moreover, *HCT1*, *HCT2*, *F5H* and *COMT* are also involved in phenolic acid biosynthesis, indicating that the genes related to phenolic acid biosynthesis were more active in RG than in BW. The expression of *HCT1*, *HCT2*, *F5H*, *COMT* and *CAD6* were suppressed under LT, indicating that LT inhibited the biosynthesis of phenolic acids and lignin.

### 3.5. Dynamics of Antioxidant Activities in Post-Harvest Chive

**Figure 6:**
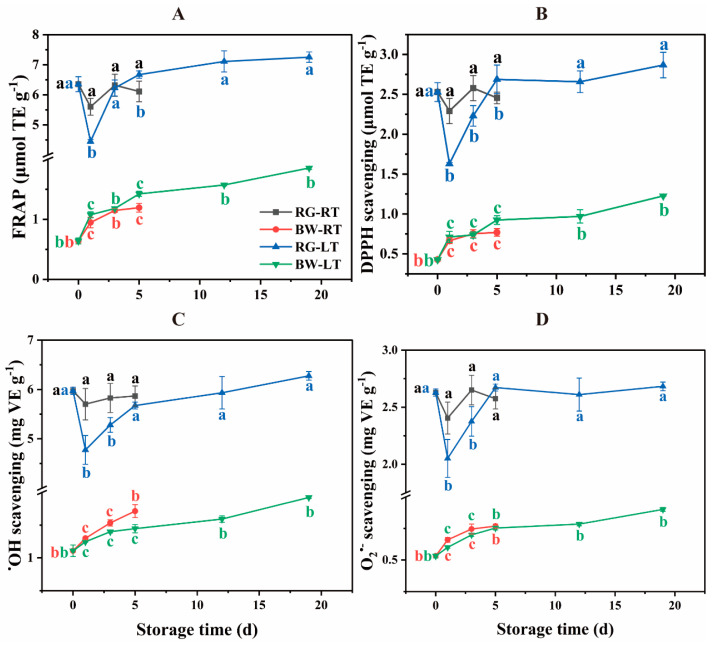
(**A**) FRAP activity, (**B**) DPPH scavenging, (**C**) ^•^OH scavenging and (**D**) O_2_^•−^ scavenging in different parts of chive stored at 20 °C (RT) and 4 °C (LT). RG, round green part. BW, bottom white part. TE, Trolox equivalents. VE, AsA equivalents. Data are presented as the means of three independent measurements. The letters represent statistical differences among different tissues under RT and LT at each time point.

As shown in Figure 6, FRAP content was 2.9- to 8.9-fold higher in RG than in BW. Moreover, in RG, they were 25.88% higher on day 1 and 8.39% lower on day 5 under RT compared with that under LT. DPPH content was 1.3- to 4.9-fold higher in RG than in BW. DPPH scavenging in RG was 40.70% and 15.56% higher on days 1 and 3, respectively, under RT than under LT, whereas it was 8.64% lower on day 5 under RT than under LT. ^•^OH scavenging was 2.3- to 4.4-fold higher in RG compared with BW. In RG, it was 19.39% and 10.35% higher under RT on days 1 and 3, respectively, than under LT. In BW, it was 18.58% higher on day 5 under RT than under LT. O_2_^•−^ scavenging was 2.0- and 3.9-fold higher in RG than in BW. Furthermore, in RG, it was 17.23% and 11.57% higher under RT than under LT on days 1 and 3. The antioxidant activities in RG slightly changed under RT and remarkably decreased on day 1, followed by a gradual increase to the initial level under LT. In contrast, the activities in BW continuously increased under both temperatures. These findings suggest that the performance of antioxidant activity in post-harvest chive differed in different tissue parts.

RG exhibited significantly higher antioxidant activity than BW. This aligns with observations of (1) significantly higher DPPH scavenging and reducing ability in the green part of leek than in the white part [6], (2) higher antioxidant potential in leaves than in bulb extracts in *Allium ursinum* L. [12] and (3) increased antioxidant activity in the aerial parts of all *Allium* species compared with the bulbs [35]. LT suppressed the antioxidant activities in RG on days 1 and/or 3 and inhibited ^•^OH scavenging in BW on day 5. This is consistent with reduced antioxidant activity in mango fruits stored at 5 °C than at 13 °C [29] and lower total antioxidant activity in eggplants stored at 1 °C than at 10 °C [36].

### 3.6. Dynamics of Other Nutrients

In our previous work [15] (Table S7), S-alk(en)ylcysteine sulfoxides (CSOs) content was higher in BW than in RG. CSOs, primarily biosynthesised in RG [16], remained largely unchanged in RG, whereas they continued to accumulate in BW. Free amino acids (FAAs) were also found to accumulate significantly in BW [15], primarily when proteolysis occurs in RG. These findings suggest that compounds for sulphur and nitrogen storage accumulated in BW in post-harvest chive.

### 3.7. Correlation Analysis

**Figure 7:**
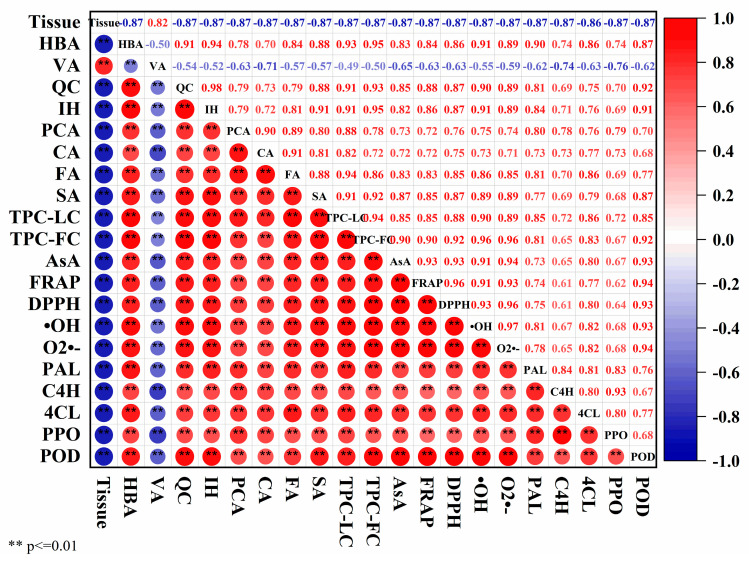
Spearman’s correlation among tissue parts, phenolic compounds, AsA, related enzymes and antioxidant parameters in different parts of chive stored at 20 °C (RT) and 4 °C (LT).

The correlation analysis is presented in Figure 7. Tissue type showed a high relationship with each parameter (|r| > 0.8 **, *p* < 0.01), indicating a correlation between parameters and tissue types. VA displayed a negative correlation with other phenolic compounds. Antioxidant activity parameters strongly correlated with HBA, QC, IH, FA, SA, TPC-LC, TPC-FC and AsA (r > 0.8 **, *p* < 0.01). This was in alignment with reports that showed increased TPC and AsA content in onions treated with licorice root extract and *Trichoderma album*, thereby enhancing antioxidant activity [37,38] and the highest phenolics coinciding with the highest DPPH scavenging and FRAP in stems compared with bulbs and flowers in *Allium pallens* L. [39]. Enzymes involved in the biosynthesis and degradation of phenolic compounds exhibited a positive correlation with each phenolic compound, TPC-LC and TPC-FC (r > 0.65 **, *p* < 0.01), except for a negative correlation with VA.

**Figure 8:**
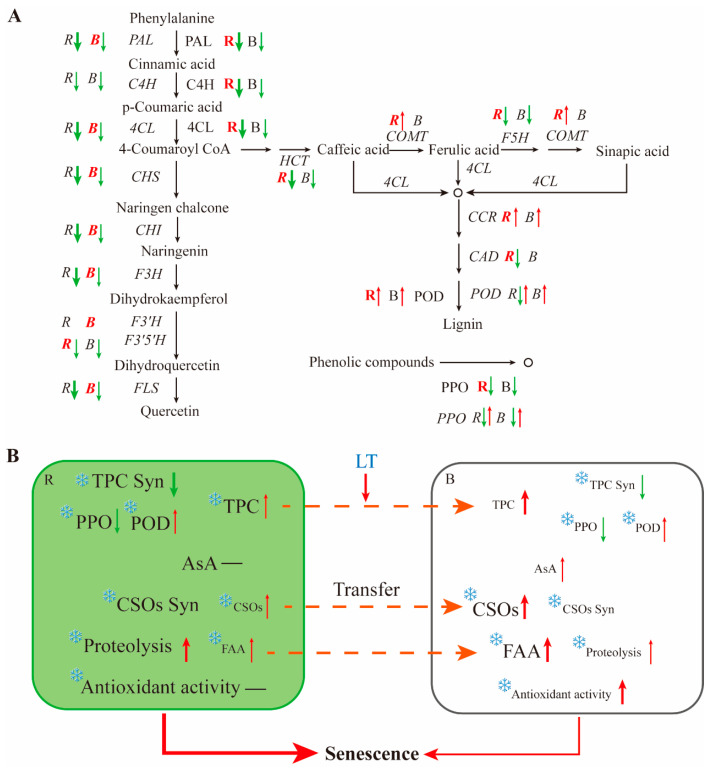
(**A**) Comparison of gene expression and enzyme activities in the phenylpropanoid pathway in different parts of post-harvest chive; and (**B**) mechanism of biosynthesis, change and transfer of different nutrients in different parts of chive during storage. *R*, round green part; *B*, bottom white part. CSOs, S-alk(en)ylcysteine sulfoxides; FAAs, free amino acids; Syn, synthesis. *R* and *B* in italics represent the expression of related genes in the corresponding part. *R* and *B* without italics represent the activity of related enzymes in the corresponding part. *R* and *B* in red denote that the tissue has higher gene expression level or enzyme activity than that observed in other tissues. ↑ means increase. ↓ means decrease. 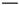 means a slight change. The thickness of the arrows indicates the extent of the change. 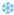 means inhibition by a low temperature.

A comparison of gene expression and enzyme activities in the phenylpropanoid pathway in different parts of the post-harvest chive is shown in Figure 8A. Enzymes related to the biosynthesis and degradation of phenolic compounds showed significantly higher activities in RG than in BW, indicating that the biosynthesis and degradation of phenolic compounds primarily occurred in RG. The activities of biosynthetic enzymes in RG remarkably declined compared with those in BW, indicating a substantial physiological change in RG. Genes related to phenolic acids and lignin biosynthesis, including *HCT*, *COMT*, *F5H*, *CCR* and *CAD*, showed higher expression levels in RG than in BW, suggesting a rapid lignin biosynthesis in RG. The expression levels of most genes related to flavonoid biosynthesis greatly decreased in RG and showed lower expression levels than in BW, indicating that BW maintained the expression of genes related to flavonoid biosynthesis.

Overall, phenolic compounds were primarily synthesised in RG, but BW maintained the expression of biosynthetic genes, the activities of biosynthetic enzymes for phenolic compounds and the expression of genes related to flavonoid biosynthesis (Figure 8B). AsA content and antioxidant activities measured using different methods gradually increased in BW, whereas they remained constant in RG. Other nutrients, such as CSOs and FAAs, were constantly transferred from RG to BW, accumulating in BW. These results suggest that RG, as the main organ for nutrient biosynthesis in the development and growth of chive, undergoes rapid senescence post-harvest. BW, as the primary storage organ, undergoes senescence much more slowly than RG. This aligns with the distinct appearance of different tissue parts in post-harvest chive.

## 4. Conclusions

To the best of our knowledge, this study represents the first investigation on the metabolism of phenolic compounds and changes in antioxidant activity in different tissues of post-harvest chive. The results show that RG exhibited significantly higher content of AsA, phenolic compounds, metabolic enzyme activities and antioxidant activities compared with those of BW, indicating that phenolic compounds were mainly synthesised in RG. The expression of related genes and enzyme activities rapidly decreased in RG, whereas they were maintained in BW, suggesting a rapid occurrence of senescence in RG than in BW. LT inhibited gene expression, enzyme activities and antioxidant activities but promoted the transfer of phenolic compounds from RG to BW. Our previous work demonstrated a constant transfer of CSOs and FAAs from RG to BW, resulting in their accumulation in BW. Combined with this finding, we can conclude that RG, as the primary organ for nutrient biosynthesis in the development and growth of chive, undergoes faster senescence post-harvest compared to BW, which was the main storage organ. The present study provides a theoretical foundation for further research and development of different parts of *Allium* plants and offers a basis for consumers’ nutritional considerations.

## Data Availability

Data is contained within the article and Supplementary Materials.

## Supplementary Materials

The following supporting information can be downloaded at: https://www.mdpi.com/article/10.3390/antiox13030279/s1, Figure S1: A representative extract of precursor-product ions chromatograph of phenolic compounds of postharvest chives; Table S1: Retention time (RT), MS/MS transitions, and parameters for phenolic compounds used in this study; Table S2: Primers used in qPCR experiments; Table S3: The calibration curves of each phenolic compounds; Table S4: TPC-FC content in different parts of postharvest chive during storage; Table S5: AsA content in different parts of postharvest chive during storage; Table S6: The content of phenolic compounds in different parts of postharvest chive during storage; Table S7: CSOs and amino acids content in different tissues of postharvest chive.
