# Supplementary material for: Metabolism of Phenolic Compounds and Antioxidant Activity in Different Tissue Parts of Post-Harvest Chive (Allium schoenoprasum L.)
Source: Antioxidants (Basel). 2024 Feb 25;13(3):279. doi: 10.3390/antiox13030279 (PMC10967344; doi:10.3390/antiox13030279)
Supplement: Supplementary file 1 [file antioxidants-13-00279-s001.zip › antioxidants-2857346-supplementary.pdf]

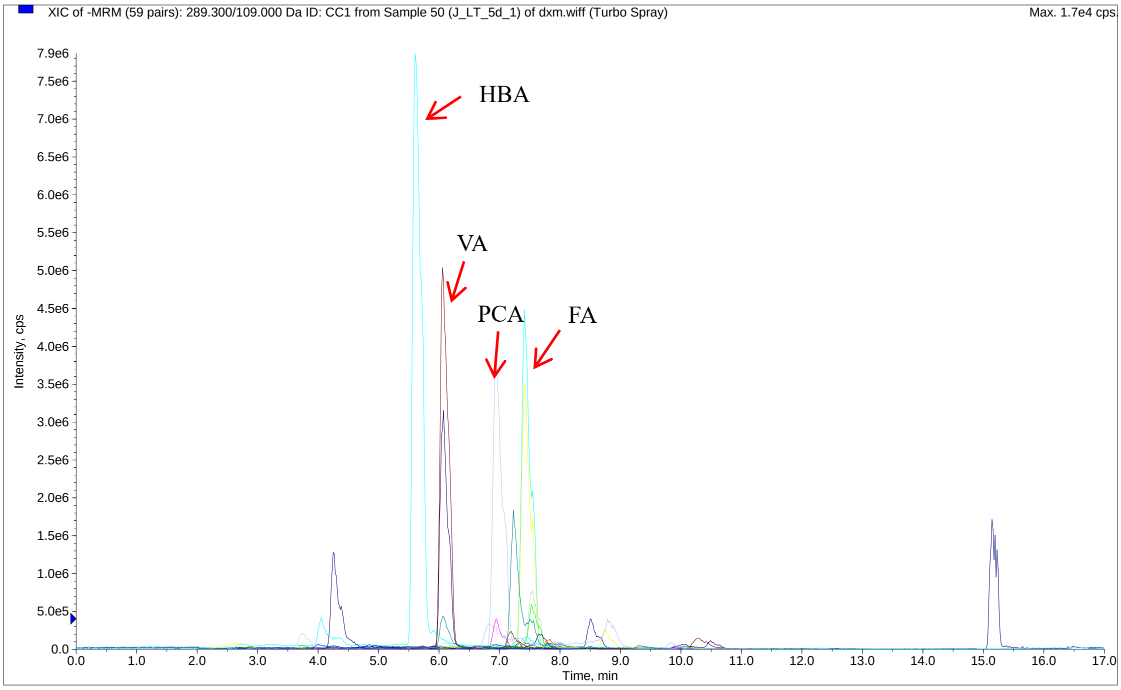

Figure S1. A representative extract of precursor-product ions chromatograph of phenolic compounds of postharvest chives. The sample came from the bottom white part of chive stored at 4 ºC for 5 days). HBA, p-hydroxybenzoic acid; VA, vanillic acid; PCA, p-coumaric acid; FA, ferulic acid.

Table S1. Retention time (RT), MS/MS transitions, and parameters for phenolic compounds used in this study

No	Compounds name	m/z $[M-H]^-$	Product ion	[a]DP (volts)	[b]CE (volts)	Retention time (min)
1	Chlorogenic acid	353.2	190.9 *	−153	−24	5.64
		353.2	178.9	−96	−36	
2	p-Coumaric acid	163.2	119.0 *	−56	−20	6.96
		163.2	92.9	−63	−38.5	
3	Caffeic acid	179.2	134.9 *	−129	−21.5	6.08
		179.2	89.2	−108	−38	
4	Ferulic acid	193.3	133.8 *	−141	−21.5	7.44
		193.3	177.9	−111	−17.5	
5	Sinapic acid	223.2	148.7 *	−123	−26.5	7.55
		223.2	193.0	−135	−28.5	
6	Isorhamnetin	315.3	255.2	−59	−22	10.33
		315.3	299.9 *	−153	−30	
7	Naringenin Chalcone	271.2	151.3 *	−129	−22	9.92
		271.2	176.8	−117	−23.5	
8	Hesperitin	301.2	216.8 *	−189	−38	13.17
		301.2	285.2	−195	−34	
9	Neochlorogenic acid	353.2	191.1 *	−111	−25	5.11
		353.2	178.8	−90	−26	
10	p-Hydroxybenzoic acid	137.1	93.0 *	−90	−19	5.64
11	trans-Cinnamic acid	147.1	103.2 *	−49	−16	9.08

		164.1	147.1 *	−102	−16.5	
12	Phenylalanine					3.74
		164.1	102.7	−110	−20	
13	Vanillic acid	167.1	123.0	−120	−18	6.09
		167.1	152.0 *	−42	−22	
14	Hyperoside	463.2	300.1 *	−180	−39	7.24
		463.2	116.9	−66	−31	
15	Gallic acid	169.2	124.8 *	−57	−19	4.01
		169.2	81.0	−90	−26	
16	Dihydroquercetin	303.1	285.1 *	−110	−18	7.45
		303.1	125.0	−135	−29	
17	Rutin	609.1	300.0 *	−184	−49	6.96
		609.1	339.1	−114	−25	
18	Luteolin	285.1	133.0 *	−194	−44	9.38
		285.1	150.9	−192	−34	
19	Quercetin	301.1	151.0 *	−163	−29	9.37
		301.1	178.9	−156	−25	
20	Apigenin	269.1	117.0 *	−165	−48	10.08
		269.1	150.9	−170	−33	
21	Dihydrokaempferol	287.2	125.2	−114	−30	8.32
		287.2	259.1	−127	−20	

Note: *Product ion for quantification. [a]DP: declustering potential, [b]CE: colloision energy

Table S2. Primers used in qPCR experiments

No	Primers name	Primers sequence
1	Actin-F	GAGCAAAGAGATTACGGCAC
2	Actin-R	CGATGAACAATGGAAGGACC
3	PAL_F	GAAACCCGAATTCACCGACC
4	PAL_R	TCGTGGAGCTTCTTTGCCAT
5	C4H1_F	AATCGAAACAACGCTATGGTC
6	C4H1_R	CAGAATTTTACTCTCAGCAGGT
7	C4H2_F	ATTATCGAGCACAAAGGCAAC
8	C4H2_R	CTATGCCCCATTCTATTGACC
9	COMT_F	TTGCCTACTGAGAATCCAC

10	COMT_R	TCTTTCAAGTAGTACCAGCTC
11	F5H_F	ACCTCGACAAACTGCCGTAC
12	F5H_R	CCACACGTTGATCATTACCCTC
13	4CL1_F	ACAGCGAATCCTTACTACACC
14	4CL1_R	TGTGTTCATCTGCCAACTCGT
15	4CL2_F	TTGCCTAATGCAGAAATTGGTC
16	4CL2_R	CTCTAATACAAATTTCCCCAGGT
17	CCR_F	TCAACCTACCGTGAATGCAAG
18	CCR_R	ACGAATATGGGCTTCAGCAAC
19	CAD6_F	TGTTATTAGCACGTCGCCCTC
20	CAD6_R	TGAATGTTGCGAACCCACT
23	CAD1_F	TATCAAGATCACGCATTGTGG
24	CAD1_R	AATAGTGGCATTCTCTACAGG
25	POD1_F	GCATCGGTACTTCTAGATGAC
26	POD1_R	GCGACAGTAAGAATATCAGC
29	POD42_F	ATACAATAAAGGAGGCAGTGG
30	POD42_R	TGGTCTGGCAAATACTTCTCC
33	CHS_F	ACGATACATGCACGTAAACGAAG
34	CHS_R	CCACTCTTTTATGGCGGCTT
35	CHI1_F	TCTATACAGACGCAGAAGCATC
36	CHI1_R	GCAATCTTTAGTGTTCCGCTTG
37	CHI3_F	TAGAATCAGCCATTAGGGACAG
38	CHI3_R	ACGTGATTACTGAACCCTT
39	F3H_F	TATCCTCTGGCAATTAGGGAAG
40	F3H_R	TTTGCCAGTTTCTTGAGCTT
41	FLS_F	TTTCATAATGTGTGGCCCAAG
42	FLS_R	TAAGTAGCTGTGCATTTTGTCC
43	F3'5'H_F	GCAGGCACAGATACAGCAAC
44	F3'5'H_R	CGGCTTGTTCCTATTACTTGGTC
45	F3'H_F	CGGTCCTTTATTTGGTCTACGTT
46	F3'H_R	TGCGAAGACTAAATCCTGGT
47	PPO1_F	TACTACATTTCCCTTCGCTCTAG
48	PPO1_R	ACCGACTTCTCTATGATCAGC
51	PPO3_F	TGGTGCTTTTCATCAAACTGG
52	PPO3_R	TAGCAGGTAAGTTCATCCCAT
53	HCT1_F	CACGTACTATCCTCACCTTGC
54	HCT1_R	TTCCGCCTCAACAAATCGAAC
55	HCT2_F	CGTCCGTAATTCCATCACCAC
56	HCT2_R	GTCCTCGGGTAATTCTCTTGC

Table S3. The calibration curves of each phenolic compounds

No	Compounds name	Equation
1	Naringenin Chalcone	$y = 8.08e^6x - 145807, R^2 = 0.9982$
2	Luteolin	$y = 1.54e^7x - 26862, R^2 =0.999$
3	Quercetin quercetin	$y = 9.75e^6x + 77820, R^2 = 0.991$
4	Phenylalanine	$y = 5.14e^6x - 124044, R^2 = 0.9992$
5	Neochlorogenic acid	$y = 3e^6x - 153098, R^2 = 0.998$
6	Hesperitin	$y = 8.21e^6x + 11309, R^2 = 0.997$
7	*p*-Coumaric acid	$y = 2.38e^7x + 107369, R^2 = 0.991$
8	Rutin	$y = 9.82e^6x + 29842, R^2 =0.998$
9	Sinapic acid	$y = 5.59e^6x + 41224, R^2 =0.993$
10	trans-Cinnamic acid	$y = 5.96e^6x + 17816, R^2 =0.995$
11	Vanillic acid	$y = 5.09e^6x + 29763, R^2 =0.996$
12	Apigenin	$y = 1.74e^7x + 52607, R^2 =0.999$
13	Caffeic acid	$y = 2.31e^7x + 205953, R^2 =0.994$
14	Chlorogenic acid	$y = 3.81e^6x + 20029, R^2 =0.993$
15	Dihydrokaempferol	$y = 4.42e^7x + 134778, R^2 =0.998$
16	Dihydroquercetin	$y = 1.66e^7x + 157810, R^2 =0.990$
17	Ferulic acid	$y = 4.92e^6x + 59838, R^2 =0.995$
18	Gallic acid	$y = 1.68e^7x - 7587, R^2 =0.995$
19	*p*-Hydroxybenzoic acid	$y = 1.76e^7x + 1080050, R^2 =0.994$
20	Hyperoside	$y = 1.50e^7x + 62155, R^2 =0.996$
21	Isorhamnetin	$y = 2.80e^7x + 5756, R^2 =0.991$

Table S4. TPC-FC content in different parts of postharvest chive during storage

Storage conditions	RT		LT	
Tissues	RG (mg GAE g^{-1})	BW (mg GAE g^{-1})	RG (mg GAE g^{-1})	BW (mg GAE g^{-1})
0 d	2.39 ± 0.029	0.19 ± 0.010	2.39 ± 0.029	0.19 ± 0.010
1 d	2.24 ± 0.127	0.32 ± 0.023	1.91 ± 0.151	0.23 ± 0.015
3 d	2.55 ± 0.118	0.50 ± 0.021	2.23 ± 0.117	0.27 ± 0.01
5 d	2.72 ± 0.082	0.58 ± 0.015	2.43 ± 0.027	0.35 ± 0.012
12 d			2.37 ± 0.132	0.51 ± 0.014
19 d			2.67 ± 0.035	0.66 ± 0.014

Note: Data are the mean of three independent measurements. TPC-FC, total phenolic content according to Folin–Ciocalteu assay. RT, 20 °C, LT, 4 °C. RG, the round green part. BW, the bottom white part. GAE, gallic acid equivalents.

Table S5. AsA content in different parts of postharvest chive during storage

Storage conditions	RT		LT	
Tissues	RG (mg g^{-1})	BW (mg g^{-1})	RG (mg g^{-1})	BW (mg g^{-1})
0 d	0.39 ± 0.018	0.08 ± 0.004	0.39 ± 0.018	0.08 ± 0.004
1 d	0.39 ± 0.022	0.13 ± 0.010	0.34 ± 0.019	0.14 ± 0.005
3 d	0.40 ± 0.020	0.16 ± 0.007	0.39 ± 0.015	0.16 ± 0.006
5 d	0.36 ± 0.018	0.17 ± 0.014	0.41 ± 0.024	0.2 ± 0.011
12 d			0.39 ± 0.012	0.23 ± 0.018
19 d			0.42 ± 0.019	0.29 ± 0.018

Note: Data are the mean of three independent measurements. AsA, ascorbic acid. RT, 20 °C, LT, 4 °C. RG, the round green part. BW, the bottom white part.

Table S6. The content of phenolic compounds in different parts of postharvest chive during storage

Tissues	Compounds name	The content of phenolic compounds (mg kg^{-1}) RT 0 d	RT 1 d	RT 3 d	RT 5 d	LT 1 d	LT 3 d	LT 5 d	LT 12 d	LT 19 d
RG	Vanillic acid	1.401±0.0259	1.270±0.0186	1.343±0.0082	1.804±0.0188	1.269±0.0356	1.327±0.0110	1.239±0.0158	1.275±0.0152	1.499±0.0274
	p-Hydroxybenzoic acid	3.209±0.0604	2.659±0.0194	2.772±0.0228	6.151±0.0936	2.257±0.0395	2.488±0.0311	2.708±0.0149	2.654±0.0656	4.445±0.0862
	Isorhamnetin	0.996±0.0339	1.174±0.0255	1.456±0.0057	1.686±0.0424	0.633±0.0156	0.888±0.0198	1.317±0.0071	1.617±0.0325	1.591±0.0948
	Quercetin	1.817±0.0095	2.919±0.0298	3.494±0.0513	3.580±0.0301	1.520±0.0102	2.007±0.0439	3.228±0.0424	3.811±0.0465	3.495±0.0413
	Ferulic acid	28.427±1.1327	27.203±0.0297	26.508±0.4639	28.786±0.4384	25.123±0.0891	22.224±0.6279	23.149±0.2644	23.813±0.0410	27.800±1.2784
	p-Coumaric acid	15.461±0.6265	14.577±0.3069	17.197±0.5643	17.391±0.1937	12.202±0.0764	11.629±0.0976	12.306±0.1838	12.553±0.6265	13.61±0.5487
	Sinapic acid	2.287±0.0072	2.595±0.0351	2.671±0.0478	2.814±0.0532	1.393±0.0144	2.221±0.0513	2.589±0.0326	2.546±0.0429	2.638±0.0230
	Caffeic acid	1.166±0.0877	1.452±0.0707	1.156±0.0481	1.405±0.0467	1.310±0.0002	0.948±0.0509	1.010±0.0311	1.155±0.0297	1.147±0.0863
	Gallic acid	0.047±0.0012	0.0414±0.0006	0.038±0.0019	0.045±0.0018	0.037±0.0007	0.037±0.0007	0.039±0.0001	0.040±0.0004	0.046±0.0005
	Chlorogenic acid	0.009±0.0007	0.005±0.0003	0.003±0.0003	0.019±0.0004	0.0002±0.0000	0.001±0.0000	0.002±0005	0.002±0.0000	0.0009±0.0000
	Luteolin	0.015±0.0012	0.002±0.0004	0.002±0.0001	0.002±0.0001	0.001±0.0002	0.001±0.0002	0.002±0.0001	0.003±0.0002	0.001±0.0001
	Naringenin Chalcone	0.114±0.0112	0.141±0.0036	0.101±0.0023	0.075±0.0026	0.117±0.0023	0.105±0.0029	0.132±0.0054	0.120±0.0025	0.086±0.0016
	Dihydroquercetin	0.023±0.0021	0.022±0.0020	0.017±0.0005	0.015±0.0002	0.024±0.0013	0.019±0.0008	0.025±0.0013	0.019±0.0007	0.016±0.0010
	Neochlorogenic acid	0.0035±0.0002	0.003±0.0002	0.003±0.0001	0.004±0.0002	0.002±0.0002	0.002±0.0001	0.002±0.0001	0.002±0.0001	0.002±0.0001

	Rutin	0.0003±0.00002	0.0003±0.00003	0.0003±0.00002	0.0001±0.00001	0.0001±0.00000	0.0001±0.00000	0.0001±0.00000	0.0002±0.00002	0.0001±0.00000
	Hyperoside	0.136±0.0091	0.224±0.0049	0.225±0.0057	0.209±0.0037	0.132±0.0016	0.170±0.0025	0.236±0.0038	0.305±0.0014	0.225±0.0015
	trans-Cinnamic acid	0.040±0.0013	0.029±0.0012	0.039±0.0007	0.026±0.0005	0.027±0.0002	0.0245±0.0006	0.025±0.0005	0.027±0.0004	0.027±0.0003
	Phenylalanine	0.0009±0.0000	0.002±0.0001	0.0127±0.0001	0.067±0.0020	0.001±0.0001	0.002±0.0001	0.009±0.0003	0.016±0.0004	0.032±0.0009
	Apigenin	0.035±0.0020	0.007±0.0002	0.007±0.0002	0.007±0.0002	0.004±0.0003	0.004±0.0001	0.006±0.0001	0.008±0.0002	0.005±0.0000
	Hesperitin	0.006±0.0003	0.006±0.0003	0.006±0.0001	0.006±0.0004	0.005±0.0002	0.005±0.0003	0.005±0.0003	0.005±0.0001	0.005±0.0001
	Dihydrokaempferol	0.050±0.0013	0.052±0.0011	0.044±0.0006	0.047±0.0010	0.060±0.0010	0.058±0.0019	0.064±0.0014	0.057±0.0018	0.058±0.0011
	Total	55.243±1.2853	54.383±0.2272	57.095±0.0530	64.139±0.597	46.120±0.1444	44.158±0.3126	48.092±0.0454	50.023±0.4947	56.727±1.3308
	**Compounds name**	**RT**				**LT**				
		**0 d**	**1 d**	**3 d**	**5 d**	**1 d**	**3 d**	**5 d**	**12 d**	**19 d**
BW	Vanillic acid	1.5883±0.00500	2.1194±0.07539	2.2860±0.04514	2.1619±0.02195	1.7596±0.02919	1.8697±0.03403	1.8908±0.01850	1.9481±0.08020	2.2510±0.01167
	p-Hydroxybenzoic acid	0.9501±0.00718	0.9766±0.00624	1.0598±0.01664	1.0362±0.01023	0.8943±0.01089	0.9249±0.01034	0.8884±0.01568	1.0261±0.00732	1.1656±0.00244
	Isorhamnetin	0.0107±0.00049	0.0321±0.00057	0.0403±0.00094	0.0500±0.00114	0.0167±0.00073	0.0195±0.00057	0.0170±0.00063	0.0226±0.00017	0.0323±0.00034
	Quercetin	0.0081±0.00105	0.0500±0.00085	0.1505±0.00165	0.0916±0.00178	0.0094±0.00023	0.0437±0.00117	0.0136±0.00041	0.0318±0.00034	0.1616±0.00094
	Ferulic acid	0.8507±0.01306	1.5282±0.02264	1.4017±0.00457	1.0914±0.00778	1.0906±0.00531	1.5050±0.02506	1.9271±0.02241	1.7630±0.02650	1.6575±0.02015
	p-Coumaric acid	0.2421±0.00254	0.3930±0.0054	0.2755±0.00232	0.2328±0.00141	0.3107±0.00452	0.3438±0.00635	0.3619±0.00356	0.2256±0.00253	0.2899±0.00259
	Sinapic acid	0.2418±0.00271	0.2974±0.00208	0.3349±0.00492	0.3083±0.00300	0.2583±0.00261	0.3103±0.00201	0.3122±0.00662	0.3420±0.00350	0.2951±0.00506
	Caffeic acid	0.0222±0.00160	0.0309±0.00111	0.0223±0.00121	0.0149±0.00182	0.0297±0.00092	0.0634±0.00200	0.0342±0.00066	0.0315±0.00173	0.0292±0.00037
	Gallic acid	0.0029±0.00006	0.0115±0.00027	0.0160±0.00038	0.0074±0.00008	0.0094±0.00048	0.0099±0.00024	0.0074±0.00015	0.0067±0.00036	0.0076±0.00034
	Chlorogenic acid	0.0578±0.00038	0.0458±0.00109	0.0152±0.00029	0.0042±0.00087	0.0036±0.00048	0.0028±0.00030	0.0038±0.00015	0.0025±0.00063	0.0022±0.00031
	Luteolin	0.0043±0.00021	0.0028±0.00001	0.0023±0.00003	0.0025±0.00006	0.0009±0.00004	0.0014±0.00008	0.0023±0.00007	0.0015±0.00011	0.0018±0.00002

Naringenin Chalcone	0.0039±0.00025	0.0027±0.00009	0.0028±0.00015	0.0030±0.00008	0.0022±0.00016	0.0019±0.00010	0.0018±0.00010	0.0019±0.00007	0.0019±0.00007
Dihydroquercetin	0.0663±0.00232	0.0579±0.00148	0.0456±0.00083	0.0385±0.00151	0.0558±0.00035	0.0530±0.00126	0.0407±0.00060	0.0361±0.00144	0.0270±0.00137
Neochlorogenic acid	0.0095±0.00069	0.0078±0.00056	0.0037±0.00029	0.0028±0.00029	0.0033±0.00010	0.00280.00018	0.0031±0.00012	0.0021±0.00013	0.0023±0.00017
Rutin	0.0016±0.00005	0.0014±0.00010	0.0006±0.00000	0.0003±0.00002	0.0001±0.00003	0.0001±0.00001	0.0001±0.00002	0.0001±0.00000	0.0001±0.00000
Hyperoside	0.0006±0.00008	0.0005±0.00008	0.0006±0.00006	0.0004±0.00004	0.0002±0.00004	0.0003±0.00002	0.0002±0.00001	0.0003±0.00003	0.0012±0.00007
trans-Cinnamic acid	0.0147±0.00048	0.0218±0.00023	0.0150±0.00038	0.0132±0.00013	0.0089±0.00016	0.0098±0.00035	0.0169±0.00048	0.0174±0.00039	0.0172±0.00066
Phenylalanine	0.0576±0.00106	0.0106±0.00065	0.0560±0.0008	0.1497±0.00253	0.0069±0.00060	0.0096±0.00104	0.0925±0.00303	0.1333±0.00246	0.1221±0.00098
Apigenin	0.0093±0.00017	0.0033±0.00001	0.0040±0.00006	0.0051±0.00012	0.0005±0.00006	0.0027±0.00006	0.0054±0.00021	0.0026±0.00008	0.0035±0.00012
Hesperitin	0.0058±0.00050	0.0054±0.00038	0.0052±0.00019	0.0051±0.00024	0.0051±0.00011	0.0053±0.00036	0.0049±0.00015	0.0053±0.00023	0.0049±0.00012
Total	4.148±0.0163	5.599±0.0870	5.742±0.0298	5.219±0.0417	4.466±0.0262	5.180±0.0382	5.624±0.0340	5.601±0.0906	6.074±0.0279

Note: Data are the mean of three independent measurements. RG, the round green part. BW, the bottom white part.

Table S7. [a] CSOs and amino acids content in different tissues of postharvest chive

Tissues	Storage time	CSOs (RT) (g kg^{-1})	CSOs (LT) (g kg^{-1})	FAA (RT) (g kg^{-1})	FAA (LT) (g kg^{-1})	SP (RT) (g kg^{-1})	SP (LT) (g kg^{-1})
RG	0 d	1.467 ± 0.043	1.467 ± 0.043	1.028 ± 0.019	1.028 ± 0.019	22.273 ± 0.069	22.273 ± 0.690
	2 d	1.348 ± 0.019	1.382 ± 0.046	1.522 ± 0.012	1.517 ± 0.020	20.914 ± 0.330	21.813 ± 0.069
	5 d	1.513 ± 0.079	1.482 ± 0.038	2.499 ± 0.086	1.907 ± 0.038	14.639 ± 0.104	22.273 ± 0.242
	12 d		1.618 ± 0.018		2.363 ± 0.011		22.592 ± 0.242
	20 d		1.633 ± 0.038		2.721 ± 0.048		21.793 ± 0.385

BW	0 d	2.330 ± 0.012	2.330 ± 0.012	1.676 ± 0.016	1.676 ± 0.016	21.173 ± 0.415	21.173 ± 0.145
	2 d	2.775 ± 0.043	2.357 ± 0.097	5.923 ± 0.081	4.077 ± 0.160	21.993 ± 0.330	22.133 ± 0.275
	5 d	2.739 ± 0.070	2.744 ± 0.142	9.097 ± 0.088	5.439 ± 0.201	22.392 ± 0.922	23.212 ±0.578
	12 d		3.047 ± 0.068		6.827 ± 0.069		23.392 ± 0.159
	20 d		3.217 ± 0.083		9.447 ± 0.137		23.032 ± 0.590

Note: a represents our previous work (Dai et al., 2022a). Data are the means of three independent measurements. RG, the round green part. BW, the bottom white part. CSOs, S-alk(en)ylcysteine sulfoxides; FAA, free amino acids; SP, soluble protein.
